# Chromatin Relaxation-Mediated Induction of p19INK4d Increases the Ability of Cells to Repair Damaged DNA

**DOI:** 10.1371/journal.pone.0061143

**Published:** 2013-04-12

**Authors:** María F. Ogara, Pablo F. Sirkin, Abel L. Carcagno, Mariela C. Marazita, Silvina V. Sonzogni, Julieta M. Ceruti, Eduardo T. Cánepa

**Affiliations:** Laboratorio de Biología Molecular, Departamento de Química Biológica, Facultad de Ciencias Exactas y Naturales, Universidad de Buenos Aires, Ciudad Universitaria Pabellón II, Buenos Aires, Argentina; Dana-Farber/Harvard Cancer Institute, United States of America

## Abstract

The maintenance of genomic integrity is of main importance to the survival and health of organisms which are continuously exposed to genotoxic stress. Cells respond to DNA damage by activating survival pathways consisting of cell cycle checkpoints and repair mechanisms. However, the signal that triggers the DNA damage response is not necessarily a direct detection of the primary DNA lesion. In fact, chromatin defects may serve as initiating signals to activate those mechanisms. If the modulation of chromatin structure could initiate a checkpoint response in a direct manner, this supposes the existence of specific chromatin sensors. p19INK4d, a member of the INK4 cell cycle inhibitors, plays a crucial role in regulating genomic stability and cell viability by enhancing DNA repair. Its expression is induced in cells injured by one of several genotoxic treatments like cis-platin, UV light or neocarzinostatin. Nevertheless, when exogenous DNA damaged molecules are introduced into the cell, this induction is not observed. Here, we show that p19INK4d is enhanced after chromatin relaxation even in the absence of DNA damage. This induction was shown to depend upon ATM/ATR, Chk1/Chk2 and E2F activity, as is the case of p19INK4d induction by endogenous DNA damage. Interestingly, p19INK4d improves DNA repair when the genotoxic damage is caused in a relaxed-chromatin context. These results suggest that changes in chromatin structure, and not DNA damage itself, is the actual trigger of p19INK4d induction. We propose that, in addition to its role as a cell cycle inhibitor, p19INK4d could participate in a signaling network directed to detecting and eventually responding to chromatin anomalies.

## Introduction

Chromatin structure is closely related to many mechanisms involving DNA such as replication, transcription, repair and recombination. As a consequence of such interaction, any event impairing the stability of chromatin is likely to compromise DNA metabolism and genome integrity. Therefore, stimuli that can drive abnormal changes in chromatin structure should be detected in order to guarantee the maintenance of the integrity and functional activity of the genomes [1], [2].

Upon exposure to DNA damaging agents, mammalian cells trigger a sequence of multi-component biochemical reactions selected to maintain genome integrity. Beyond the activation of DNA repair enzymes, the DNA damage response (DDR) includes a complex system of signaling molecules which activate different cellular processes such as cell cycle checkpoints, apoptosis and transcription of specific target genes [3], [4], [5]. However, a cell may fail to repair the damage, in which case, genome stability becomes compromised and this may eventually lead to oncogenesis [6], [7], [8].

There is growing evidence that, when DNA damage is generated, chromatin suffers structural changes around the lesion. These changes not only take place at the level of the surrounding histones [9], [10], but also at a more global level, as it happens with the phosphorylation of the core histone variant H2AX which extends many kilobases away from each double-strand break [11], [12]. DNA damage actually seems to elicit a wide-range phenomenon according to some reported examples as is the case of p53 which, in association with the acetyl transferase p300, has been shown to mediate global chromatin decondensation after UV irradiation [13]. Moreover, some reports support the notion that chromatin structure reorganization, even in the absence of DNA damage, may be enough to activate DDR in the cell [14]. This raises the question of whether DNA damage might be a mere initiator of changes in chromatin organization, the latter being the actual trigger of some of the events elicited during the DDR in the cell. In fact, chromatin remodeling following DNA double strand break has been demonstrated to be important in the signaling and dynamics of the DNA repair machineries [15].

INK4 proteins are a family of cell cycle inhibitors that bind to CDK4/6 kinases and thus block the G1 to S phase transition of the cell cycle [16], [17], [18]. There are several experimental results supporting that one of them, p19INK4d (hereafter referred as p19), also participates in DNA damage repair, and this new role seems to be independent of its capacity to regulate cell cycle progression [19], [20]. Interestingly, p19 expression is induced upon diverse genotoxic insults and this induction is specific for this INK4 member only [19], [21]. The introduction of damaged DNA molecules into the cell has been reported to induce some DNA damage responsive genes by mimicking the effects of UV irradiation, as is the case of p21Cip1, a member of the Cip/Kip family of cell cycle inhibitors [22]. However, when damaged DNA molecules are introduced into the cell (either oligonucleotides or plasmids), p19 induction is not observed, suggesting that the presence of DNA damage is not enough to induce p19 transcription [21].

These data allow us to hypothesize that p19 induction after genotoxic insults might indeed be a consequence of the chromatin structural alterations that take place when the damage is generated. Supporting this idea, in this work we demonstrated that chromatin relaxation elicited the induction of p19 and, interestingly, that this occurred in the absence of DNA damage. We also found that this induction was dependent upon ATM/ATR, Chk1/Chk2 and E2F activity, both after treating cells with DNA damaging agents and when chromatin structure was altered, pointing at a common pathway for p19-stimulated induction. In addition, p19 improved DNA repair when the genotoxic injury was caused in a relaxed-chromatin context. The distinct response of different genes to the presence of exogenous damaged DNA [21], along with the evidence presented here, strongly supports the existence of different mechanisms involved in cellular DDR. These mechanisms would be specifically activated in response to different stimuli such as DNA damage itself or chromatin structural changes, which then eventually lead to the onset of certain sets of genes depending on the type of stimulus received.

We propose that, in addition to its role as a cell cycle inhibitor, p19 could participate in a signal network directed to detecting and eventually responding to chromatin anomalies.

## Results

### p19 is Induced by Chromatin Relaxation

When DNA damage is generated in a cell, the lesion elicits a cascade of both physical and biochemical events, which ultimately causes a change in chromatin structure not only around the damage but also many kilobases away from the lesion and even at a global scale in the whole genome [11], [13]. Given that transfected damaged DNA oligonucleotides, which lack chromatin organization, do not suffice to trigger p19 induction, we speculated that the downstream chromatin reorganization following the appearance DNA damage might be the actual initiator signal leading to the induction of the p19 gene.

**Figure 1:**
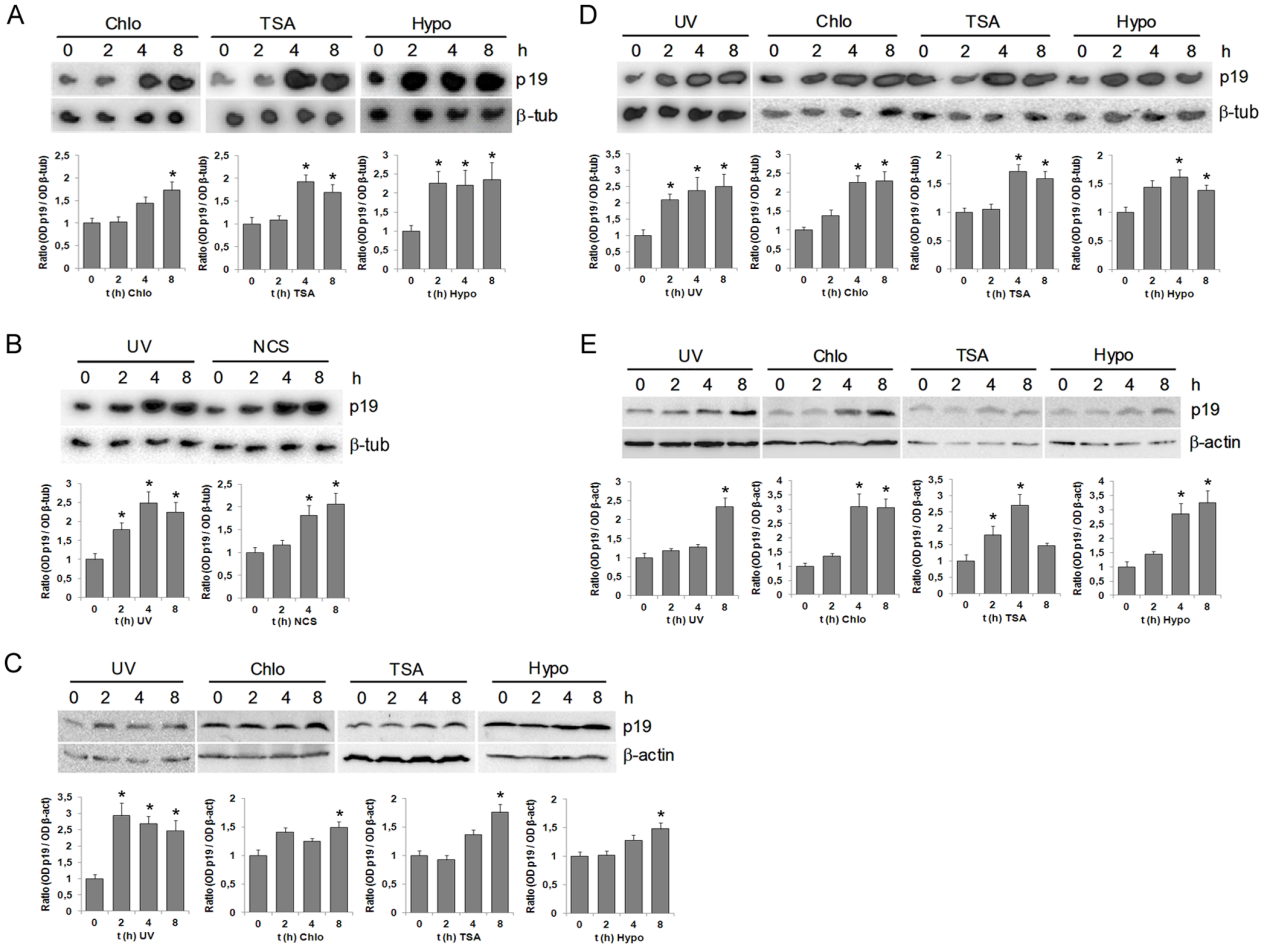
Chromatin relaxation triggers the induction of p19. **A.** HEK-293 cells were incubated with 100 µM chloroquine or 200 nM TSA or hypotonic medium (50 mM NaCl) as indicated. Total RNA (10 µg) extracted from cells at the indicated times were subjected to northern blot analysis with the ^32^P-labeled probes specified at the right margin. **B.** HEK-293 cells were irradiated with 40 J/m^2^ or incubated with 50 ng/ml NCS. The expression of p19 was assessed by northern blot. **C** and **E.** HEK-293 cells (**C**) or SH-SY5Y cells (**E**) were irradiated with 40 J/m^2^ UV or incubated with 100 µM chloroquine or 200 nM TSA or hypotonic medium and cell lysates prepared at the indicated times. Western blot analysis of p19 were carried out with 20 µg of total cellular proteins and detected with p19 monoclonal antibody. Anti-β-actin antibody was uses as a protein loading control. **D.** SH-SY5Y cells were irradiated with 40 J/m^2^ UV or incubated with 100 µM chloroquine or 200 nM TSA or hypotonic medium (50 mM NaCl) as indicated. Total RNA (10 µg) extracted from cells at the indicated times were subjected to northern blot analysis with the ^32^P-labeled probes specified at the right margin. In all cases (**A**–**E**) results are representative of three independent experiments with similar results. Densitometric analysis of p19 is represented in the lower panels. Bars represent the mean ± S.D. of three experiments. Student’s *t*-test was used to compare samples obtained at different times with samples obtained at zero time (* p<0.05, at least). Chloroquine (chlo), hypotonic medium (hypo), β-tubulin (β-tub), neocarzinostatin (NCS).

We first analyzed whether an alteration in chromatin structure might by itself trigger p19 gene induction. To answer this question, we induced global chromatin relaxation in the cells by using three well-documented chromatin-modifying agents: chloroquine, trichostatin A (TSA), and hypotonic medium [23]. The effect of these agents on chromatin condensation was evaluated by digestion with micrococcal nuclease, which preferentially cuts the DNA in the linker region between nucleosomes. All the treatments assayed caused a marked increase in cellular chromatin accessibility to micrococcal nuclease (Fig. S1). When cells were incubated with 100 µM chloroquine, 200 nM TSA or medium containing 50 mM NaCl (representing a hypotonic condition), p19 expression was induced (Fig. 1A). Interestingly, the levels and kinetics of p19 induction obtained with these chromatin-modifying agents were similar to those obtained when cells were irradiated with 40 J/m^2^ UV or incubated with 50 ng/ml neocarzinostatin (Fig. 1A and B). We also observed induction of p19 protein after incubation with each of the chromatin modifiers (Fig. 1C). A similar increase in both mRNA and protein expression of p19 was observed in human neuroblastoma SH-SY5Y cells irradiated with UV or treated with chloroquine, TSA or incubated in hypotonic conditions (Fig. 1D and E).

It has been previously reported that DNA damage triggers the induction of p19 but not of the other INK4 proteins [19], [20]. So, we speculated that chromatin modifications, as a downstream effect of DNA damage according to our hypothesis, should also have a specific effect over p19 but not over the other INK4 variants. In fact, mRNA expression analysis showed that p16INK4a, p15INK4b, and p18INK4c are not induced when cells are subjected to any of the chromatin-modifying conditions tested (Fig. S2). The parallelism observed between the effects of DNA damage and chromatin modification over the INK4 family members strengthens our idea of how the cell responds to DNA damage and points to chromatin modifications as an intermediate step in this process.

The above results suggest that chromatin structure alterations caused by chloroquine, TSA or hypotonic medium were sufficient to induce p19 expression. However, as p19 mRNA levels are also increased by several genotoxins, we tested whether those treatments could cause DNA damage. To rule out this possibility, we analyzed the phosphorylation status of H2AX, a histone variant that is present in chromatin and that is phosphorylated around double-strand breaks, constituting an indicator of DNA damage in the cell [24]. When cells were incubated with chlroquine, TSA or hypotonic medium, γH2AX remained undetected (Fig. S3A), suggesting that the induction observed was actually due to chromatin modifications and not a product of the DNA damage generated as a side-effect of the treatments applied. As a positive control, addition of the damaging agent camptothecin [25] induced H2AX phosphorylation (Fig. S3A). Furthermore, unlike irradiation with UV light, none of the treatments used to cause alterations in chromatin structure were able to form cyclobutane pyrimidine dimers (CPDs) in Neuro-2a cells (see below and data not shown). Moreover, it is very unlikely that the three chromatin modifiers assayed, which are structurally and functionaly unrelated, would induce DNA damage other than double strand-breaks or CPD dimers.

It is known that p19 expression fluctuates with cell cycle progression and that this protein accumulates during G1/S transition and S phase to fall again through the remainder of the cycle [19], [26]. A chance exists that these treatments might somehow favor the arrest of cells at G1/S phases, giving rise to an indirect increase of p19 expression, obscuring the actual effect of chromatin alteration in p19 gene induction. To explore this possibility, we performed flow-cytometric analysis to determine the cell cycle distribution of cells subjected to chloroquine, TSA or hypotonic conditions. None of the chromatin-modifying conditions evaluated had any effects on cell cycle distribution (Fig. S3B). Conversely, flow cytometric analysis 24 h following incubation with 500 nM mimosine, a ribonucleotide reductase inhibitor, arrested cells at G1/S boundary. These results indicate that alterations in chromatin structure trigger a cell response that eventually leads to p19 induction, which is independent of DNA damage and cell cycle progression.

Other authors have reported that TSA induces formation of H2AX gamma foci in a way similar to genotoxic DNA damage [27], [28] and that it changes the distribution of cells in the different phases of cell cycle [29]. However, those effects were observed between 12 and 24 h after TSA treatment and at larger concentration than that used in this work. So, our results cannot be compared with those reported in those papers. We do not discard that alterations in the cell cycle or H2AX phosphorylation by the chromatin modifiers used may happen latter in our system, but are not related to the prior induction of p19. On the other hand, Baure et al. [30] studied the changes in chromatin structure caused by incubation in a hypotonic medium, and like that observed in our work, found no changes in the cell cycle profile. However, they detected H2AX phosphorylation after 1 h of treatment in hypotonic medium.

### Chromatin Relaxation Induces p19 through the ATM Signaling Pathway

**Figure 2:**
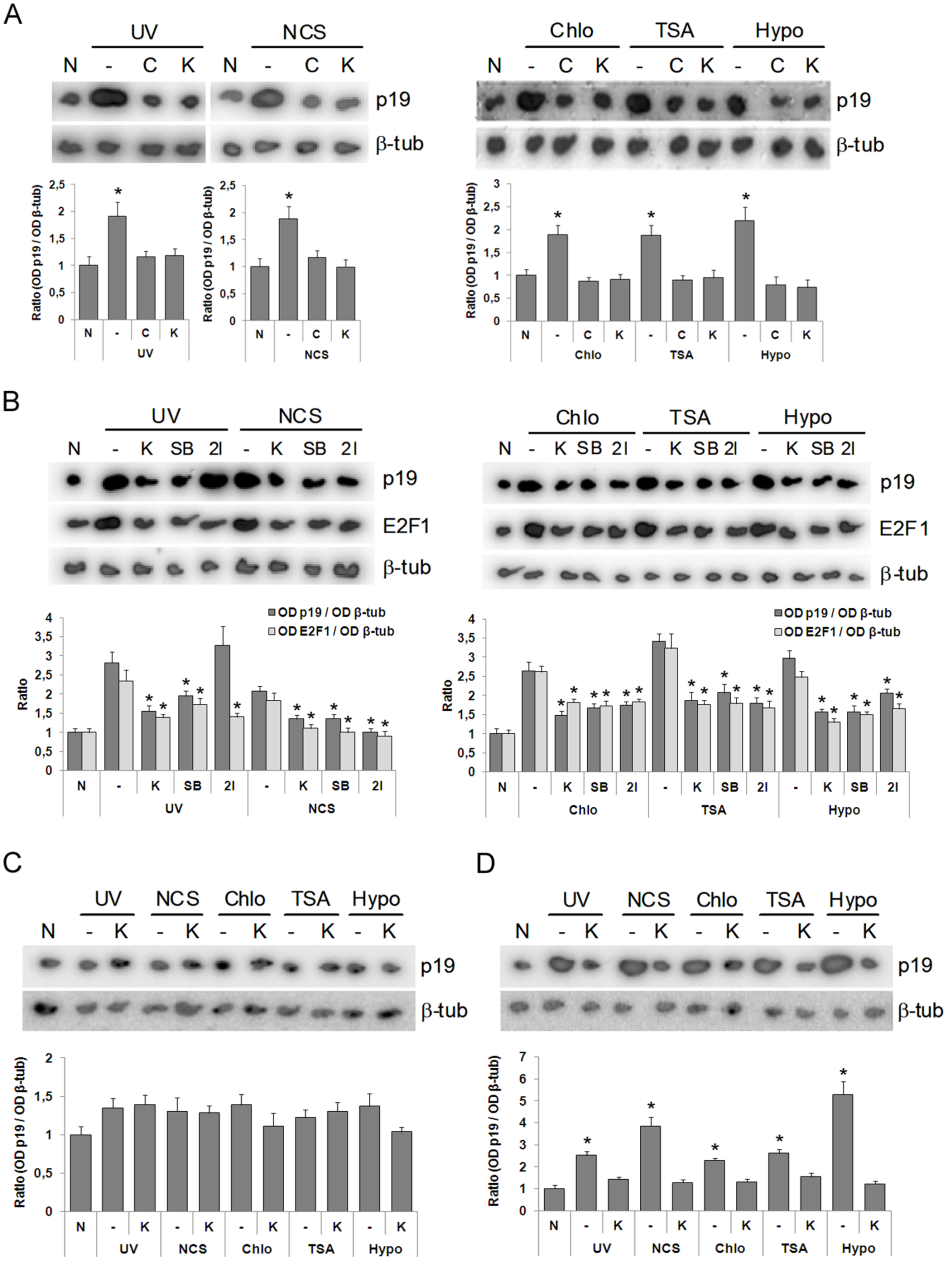
DNA damage or chromatin relaxation induces E2F1 and p19 through ATM/ATR-Chk1/Chk2 signaling. **A.** HEK-293 cells, previously treated with 10 µM Ku-55933 or 5 mM caffeine for 1 h, were exposed to 40 J/m^2^ UV or 50 ng/ml neocarzinostatin (left panel) or incubated with 100 µM chloroquine or 200 nM TSA or subjected to hypotonic medium (right panel). **B.** HEK-293 cells, previously treated with 10 µM Ku-55933 or 15 nM SB-218078 or 20 nM Chk2 inhibitor for 1 h, were exposed to 40 J/m^2^ UV or 50 ng/ml neocarzinostatin (left panel) or incubated with 100 µM chloroquine or 200 nM TSA or subjected to hypotonic medium (right panel). In (**A**) and (**B**) after 4 h, cells were harvested and subjected to northern blot analysis using a ^32^P-labelled probe specific for human p19 mRNA and reprobed for E2F1 and β-tubulin mRNA. **C** and **D.** ATM-deficient Seckel cells (**C**) or primary human fibroblasts C5RO (**D**), previously treated with 10 µM Ku-55933, were exposed to 40 J/m^2^ UV or 50 ng/ml neocarzinostatin or incubated with 100 µM chloroquine or 200 nM TSA or subjected to hypotonic medium (50 mM NaCl). In (**C**) and (**D**) after 4 h, cells were harvested and subjected to northern blot analysis using a ^32^P-labelled probe specific for human p19 mRNA and reprobed for β-tubulin mRNA. Each figure shows a representative autoradiograph of three independent experiments with similar results. Densitometric analysis of p19 and E2F1 are represented in the lower panels. Bars represent the mean ± S.D. of three experiments. Student’s *t*-test was used to compare treated and non-treated samples (* p<0.05, at least). None (N), β-tubulin (β-tub), caffeine (C), Ku-55933 (K), chloroquine (chlo), hypotonic medium (hypo), SB-218078 (SB), Chk2 inhibitor (2I).

To further support these observations, we deepened into the signaling cascade behind both scenarios: DNA damage and chromatin modification. ATM, a phosphatidyl 3-kinase-like kinase, a well-documented kinase involved in DDR, has also been found to be activated under chromatin disturbing conditions such as the ones used herein [14]. We therefore aimed at this kinase in a first attempt to decipher the signaling elements involved after DNA damage and chromatin modification. Preincubation of cells with Ku-55933, a specific inhibitor of ATM [31], as well as preincubation with caffeine, a broad-range inhibitor of phosphatidyl 3-kinase-like kinases [32], abolished p19 induction not only when the cells were exposed to a DNA-damaging agent (Fig. 2A, left panel), but also when chromatin was distorted by chloroquine, TSA or hypotonic medium (Fig. 2A, right panel). This indicates that p19 induction is dependent upon ATM activation. So far, both stimuli were shown to be signaled by the same kinase, supporting the notion that changes in chromatin structure might be an immediate downstream event leading to p19 induction after DNA damage. We next analyzed if ATR, another phosphatidyl 3-kinase-like kinase involved in DNA damage response, was required for p19 induction after genotoxic treatment or chromatin alteration. To do this, we performed similar experiments in Seckel cells that display impaired phosphorylation of ATR-dependent substrates [33]. Neither genotoxins nor chromatin modifiers were able to induce p19 in ATR-deficient Seckel cells (Fig. 2C). Conversely, UV, neocarzinostatin and incubation under the three chromatin disturbing conditions tested increased the levels of p19 mRNA in primary human fibroblasts C5RO, an induction that was abolished when cells were preincubated with Ku-55933 (Fig. 2D). These results imply that both ATM and ATR kinases are involved in p19 induction.

It has been previously shown that TSA treatment triggers p19 induction by a *cis* acting mechanism [34], so this far, the effect of TSA on p19 might actually be due to a simple effect of this drug over p19 own promoter. However, it is important to notice that, given that ATM and ATR inhibition abrogated p19 induction under all the above-mentioned conditions, the possibility of an effect in *cis* of TSA on p19 promoter should be discarded, at least as the main reason, because p19 induction needed intermediate factors, in this case ATM and ATR.

To further explore the molecular events leading to p19 induction after DNA damage and to better understand the role of chromatin in this process, we studied the role of two downstream kinases that are activated by ATM: Chk1 and Chk2 [6]. Specific Chk1 and Chk2 inhibitors, SB-218078 and 2I respectively, blocked p19 induction under the three chromatin-disturbing conditions tested, indicating that both Chk1 and Chk2 are necessary for p19 gene induction when chromatin relaxation is induced (Fig. 2B, right panel). This also showed to be the case when DNA damage was triggered by neocarzinostatin (Fig. 2B, left panel). However, it is interesting to notice that only Chk1 seems to be necessary for p19 induction when cells are exposed to UV damaging conditions. A possible explanation for this observation is that, given its wide spectrum of effects in a cell, UV might be activating a Chk2 alternative signaling pathway that somehow compensates the lack of Chk2 when this kinase is inhibited.

### Specific Induction of p19 by Chromatin-relaxing Agents

**Figure 3:**
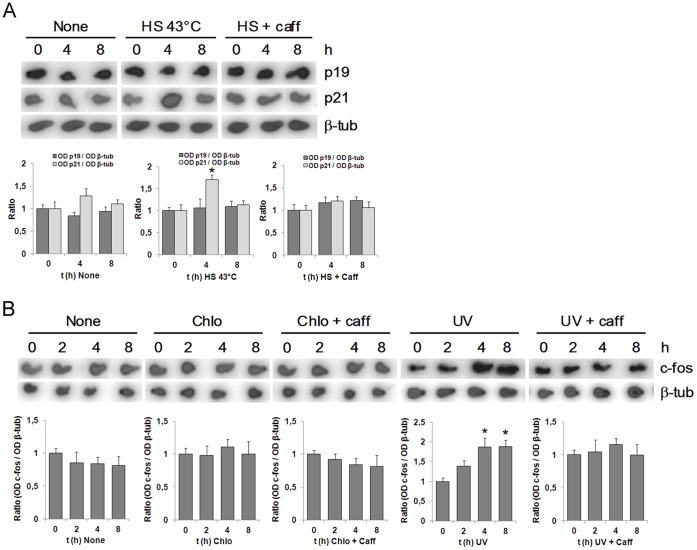
Chromatin relaxation-mediated induction of p19 is specific. **A.** HEK-293 cells, previously treated or not with 5 mM caffeine during 1 h, were incubated at 43°C for 1 h and then cultured at 37°C from 0 to 8 h. **B.** HEK-293 cells, previously treated with 5 mM caffeine for 1 h, were incubated with 100 µM chloroquine at the indicated times. In (**A**) and (**B**), cells were harvested and subjected to northern blot analysis using a ^32^P-labelled probe specified at the right margin. Each figure shows a representative autoradiograph of three independent experiments with similar results. Densitometric analysis of p19, p21 and c-fos are represented in the lower panels. Bars represent the mean ± S.D. of three experiments. Student’s *t*-test was used to compare samples obtained at different times with samples obtained at zero time (* p<0.05, at least). β-tubulin (β-tub), heat shock (HS), caffeine (caff), chloroquine (chlo).

The results described so far indicate that p19 induction, whether by genotoxin or by chromatin-remodeling agents, is mediated by ATM. This kinase becomes activated in response to a great variety of stress stimuli and participates in numerous signal transduction pathways [5], [35]. We therefore sought to examine whether the effect of the chromatin remodeling agents on p19 was specific, or if, in contrast, any stimulus capable of activating ATM would also induce p19. Since ATM is also activated by heat shock, which occurs independently of DNA damage [36], we analyzed the effect of this treatment on p19 expression. We observed that p19 levels remained unaffected by heat shock until at least 8 h following treatment (Fig. 3A). In contrast, p21 mRNA levels were upregulated 4 h after treatment, as previously reported [37], and this induction was prevented by caffeine.

To further explore the specificity of this effect, we asked whether chromatin-remodeling agents are able to induce the expression of other ATM-regulated genes. We therefore examined the expression of c-fos, a gene positively regulated by ATM in response to DNA damage [38]. Whereas both UV irradiation and neocarzinostatin treatment activated c-fos transcription, chloroquine treatment did not (Fig. 3B).

Taken together, these results show that p19 induction, caused by chromatin-remodeling agents and mediated by ATM, is a specific event. This conclusion, along with the fact that other genes were not induced under these conditions, as is the case for the other INK4 proteins analyzed, indicates that p19 induction is a specific and downstream-regulated event after chromatin remodeling.

### Chromatin Relaxation needs the E2F1 Transcription Factor for p19 Induction

**Figure 4:**
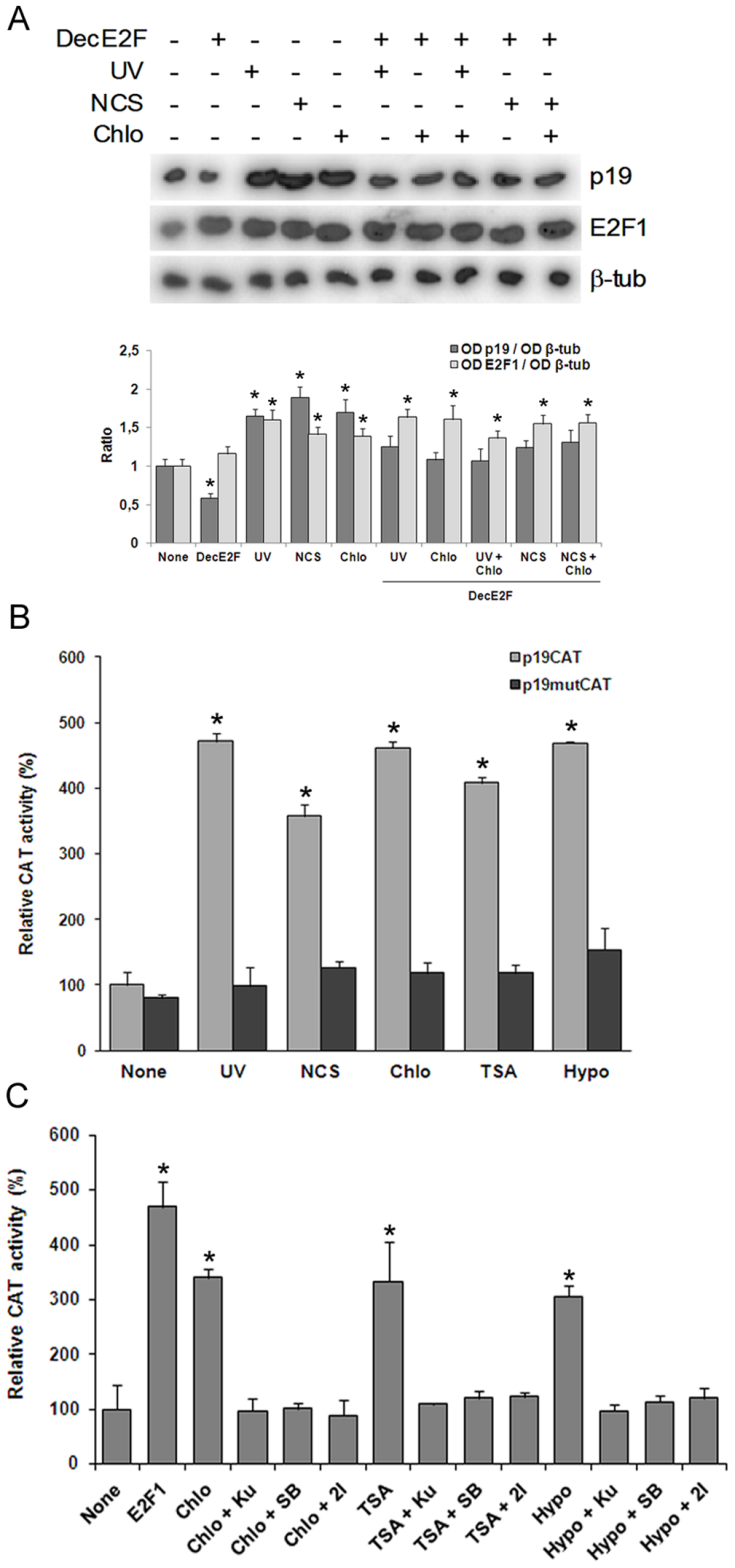
E2F mediates induction of p19 in response to DNA damage or chromatin relaxation. **A.** HEK-293 cells were transfected with 500 nM E2F decoy oligonucleotide. Twenty four hours later, cells were exposed to 40 J/m^2^ UV or 50 ng/ml NCS and incubated in the presence or in the absence of 100 µM chloroquine. After 4 h, cells were harvested and subjected to northern blot analysis using a ^32^P-labelled probe specific for human p19 mRNA and reprobed for E2F1 and β-tubulin mRNA. Figure shows a representative autoradiograph of three independent experiments with similar results. Densitometric analysis of p19 and E2F1 are represented in the lower panel. Bars represent the mean ± S.E of three experiments. Student’s *t*-test was used to compare treated and non-treated samples (* p<0.05, at least). **B.** HEK-293 cells transiently cotransfected with 4 µg of p19CAT, or equivalent amount of mutant plasmid, containing the 5′-flanking region of p19 gene and 5 µg pCEFL-β-galactosidase were exposed to 40 J/m^2^ UV or incubated with 50 ng/ml NCS or 100 µM chloroquine or 200 nM TSA or hypotonic medium. After 24 h cells were harvested and CAT activity was determined as described. Results are expressed as relative CAT activity with respect to basal value of p19CAT which was set to 100. Bars represent the mean ± S.D. of three independent experiments performed in quadruplicate. Student’s *t*-test was used to compare treated with non treated samples (* p<0.01). **C.** HEK-293 cells, transiently cotransfected with 4 µg of pE2F4XCAT and 5 µg pCEFL-β-galactosidase and, when indicated, 4 µg of a vector expressing E2F1 cDNA, were treated with 100 µM chloroquine, or 200 nM TSA or subjected to hypotonic medium and incubated in the presence or in the absence of 10 µM Ku-55933 or 15 nM SB-218078 or 20 nM Chk2 inhibitor. After 24 h cells were harvested and CAT activity was determined as described. Results are expressed as relative CAT activity with respect to basal value of pE2F4XCAT which was set to 100. Bars represent the mean ± S.D. of three independent experiments performed in quadruplicate. Student’s *t*-test was used to compare treated with non treated samples (* p<0.01). Decoy E2F oligonucleotide (DecE2F), β-tubulin (β-tub), chloroquine (chlo), hypotonic medium (hypo), neocarzinostatin (NCS), Ku-55933 (Ku), SB-218078 (SB), Chk2 inhibitor (2I).

Previous results from our lab have shown that p19 induction triggered by UV irradiation is mediated by the transcription factor E2F1 (Fig. 4A). In order to analyze whether p19 induction elicited by chromatin relaxation is also E2F-dependent, we tested the cells in the presence of a decoy oligonucleotide harboring the E2F consensus binding site. As was the case for UV, chloroquine-triggered p19 induction showed to be dependent upon E2F, and this was also the case for neocarzinostatin damage (Fig. 4A).

To confirm the functional contribution of E2F1 factors to the regulation of p19 transcription by chromatin relaxation, we constructed a reporter plasmid harboring 2250 bp of the 5′-flanking region of the p19 gene. This region contains two functional E2F-binding sites responsible for the genotoxin-mediated induction of p19 located at −685 and −636 from the translation initiation site [39]. HEK-293 cells were transiently transfected with this p19CAT vector and then incubated with each of the chromatin-modifying agents or treated with neocarzinostatin or UV irradiated before harvesting and analysis of chloramfenicol acetyltransferase (CAT) activity. Chloroquine, TSA and hypotonic medium induced p19CAT expression comparable to that observed with genotoxins (Fig. 4B). The effect of the same treatments on the transcriptional activity of the p19 gene promoter was almost completely blocked in mutant-carrying changes in both E2F1 binding sites, proving that, as is the case for genotoxins, p19 induction by chromatin-relaxing agents needs the E2F1 transcription factor and functional binding sites in its promoter.

These results led us to hypothesize that E2F1 might be the molecule that mediates the effects of both events (DNA damage and alteration in the chromatin structure) on the expression of the p19 gene across the ATM/ATR-Chk1/Chk2 pathway. Then, we analyzed whether the expression and/or transcriptional activity of E2F1 is affected by genotoxic agents and by the treatments that modify chromatin structure. The expression of E2F1 was induced in cells exposed to UV light or treated with neocarzinostatin (Fig. 2B). A similar induction of E2F1 was observed when the cells were incubated with TSA or chloroquine or cultured in a hypotonic medium. In addition, in both cases, the induction of E2F1 expression was blocked almost totally by incubation with an inhibitor of ATM or with inhibitors of Chk1 or Chk2 (Fig. 2B). These results suggest that a signal transduction pathway, common between both events (the induction of p19 and E2F1), is activated after the treatment with chromatin-remodeling agents.

We next asked whether this increase in E2F1 expression correlated with an increase in its transcriptional activity. To test this, HEK 293 cells were transfected with a plasmid containing a minimal promoter with four consensus sites for the binding of E2F transcription factors upstream the CAT reporter gene and then incubated with the remodeling agents of chromatin. The treatment with each of them caused an increase in the CAT activity higher than 3-fold (Fig. 4C).This increase in CAT activity was suppressed when the cells were pre-incubated with the inhibitor of ATM or with the inhibitors of Chk1 or Chk2. Together, these results support our hypothesis that E2F1 would operate downstream Chk1 and Chk2 in the signal pathways that mediate p19 induction in response to changes in chromatin structure.

### Chromatin Relaxation and DNA Damage Share the Same Signaling Pathway

In an attempt to confirm that chromatin relaxation is a downstream event in the signaling cascade triggered by DNA damage that results in p19 induction, we decided to treat cells with p19 inducing conditions, i.e. chromatin relaxation and DNA damage, at the same time. If our hypothesis was correct, chromatin relaxation downstream events would be shared by both these triggering agents and, under saturating conditions [19], no additive or synergistic effect should be seen in cells stimulated with both p19 inducing conditions.

**Figure 5:**
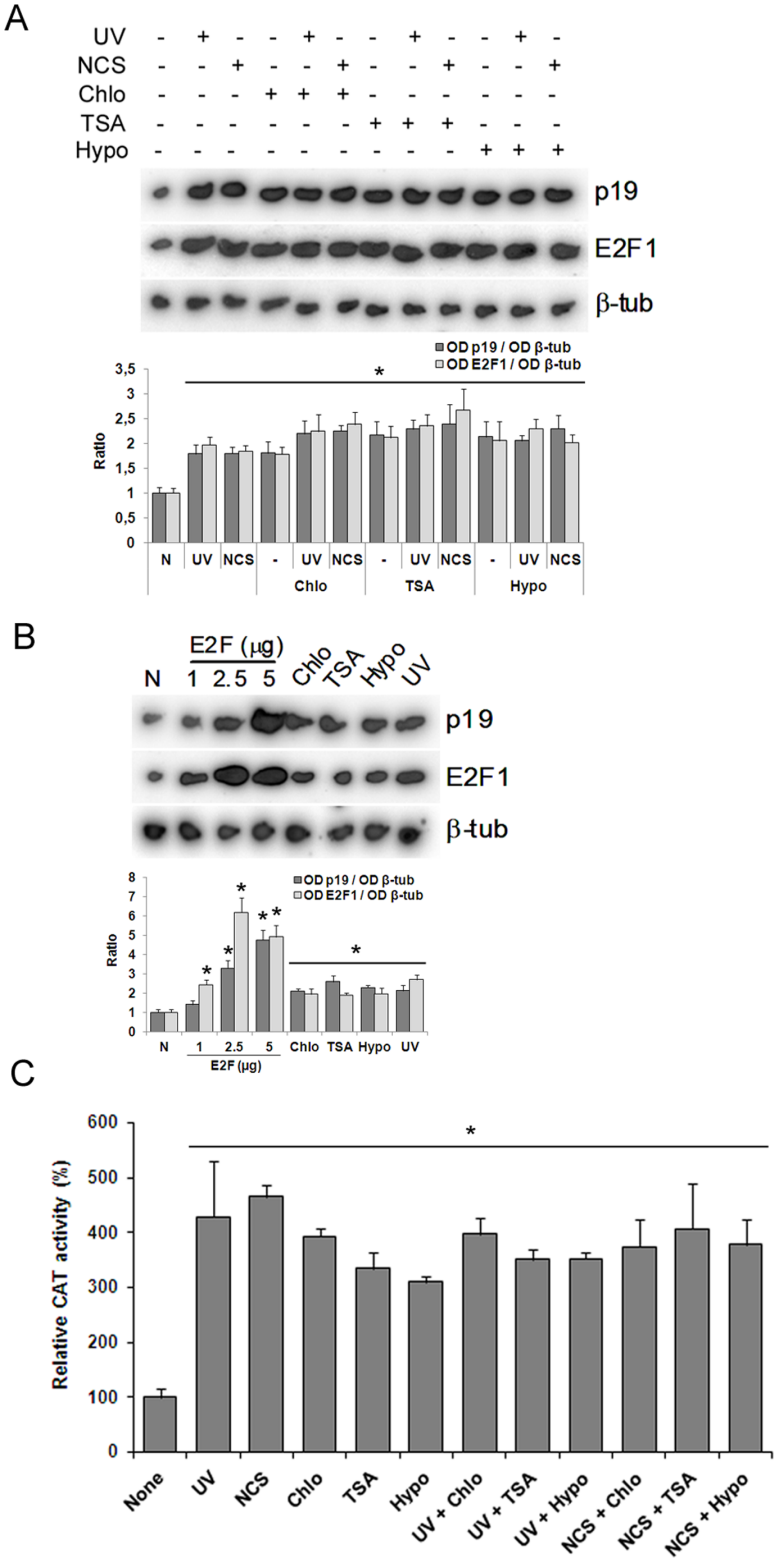
Absence of synergism of genotoxins and chromatin modifiers effects on p19 induction. **A.** HEK-293 cells were exposed to 40 J/m^2^ UV or 50 ng/ml neocarzinostatin and incubated in the presence or in the absence of 100 µM chloroquine, or 200 nM TSA or hypotonic medium. After 4 h, cells were harvested and subjected to northern blot analysis using a ^32^P-labelled probe specific for human p19 mRNA and reprobed for E2F1 and β-tubulin mRNA. **B.** HEK-293 cells were transfected with increasing levels of an expression vector encoding E2F1 gene, harvested after 24 h, and p19 and E2F1 expression assessed by northern blot. In parallel cells were incubated with 100 µM chloroquine or 200 nM trichostatin A or hypotonic medium or exposed to 40 J/m^2^ UV as indicated. After 4 h p19 and E2F1 expression was determined by northern blot. In (**A**) and (**B**) each figure shows a representative autoradiograph of three independent experiments with similar results. Densitometric analysis of p19 and E2F1 are represented in the lower panels. Bars represent the mean ± S.D. of three experiments. Student’s *t*-test was used to compare treated and non-treated samples (* p<0.05, at least). **C.** HEK-293 cells, transiently cotransfected with 4 µg of p19CAT and 5 µg pCEFL-β-galactosidase, were exposed to 40 J/m^2^ UV or treated with 50 ng/ml NCS and incubated in the presence or in the absence of 100 µM chloroquine or 200 nM TSA or hypotonic medium. After 24 h cells were harvested and CAT activity was determined as described. Results are expressed as relative CAT activity with respect to basal value of p19CAT which was set to 100. Bars represent the mean ± S.D. of three independent experiments performed in quadruplicate. β-tubulin (β-tub), chloroquine (chlo), neocarzinostatin (NCS), hypotonic medium (hypo).

As described above, cells damaged with UV or neocarzinostatin showed an increase in p19 mRNA levels similar to that in cells treated with the chromatin-relaxing agents (Fig. 1A and B and Fig. 5A). Notably, when cells were exposed to both situations simultaneously, no increases in p19 mRNA levels or p19 promoter transcriptional activity were observed with respect to the levels obtained with one condition alone, as would have been expected if DNA damage and chromatin relaxation had been triggering different signaling cascades (Fig. 5A and C). High concentrations of E2F revealed that p19 induction can reach higher levels than those obtained by any of the conditions tested in the present work, indicating that additive or synergistic effects should have been seen if they had existed, given that the transcriptional response of p19 promoter was not saturated (Fig. 5B).

This result strengthens the idea that UV might be actually activating a Chk2 alternative kinase, because the lack of additive or synergistic effects (in cells treated with UV and one of the chromatin-relaxing agents) can only be explained if both stimuli trigger the induction of p19 through the same cascade of kinases or at least through redundant kinases.

Following the same criterion, we exposed cells to a combination of DNA damage and chromatin relaxation in the presence of E2F decoy molecules. No differences were observed between damaged cells, chromatin relaxed cells and cells subjected to both conditions together (Fig. 4A), again pointing at a common pathway between DNA damage and chromatin relaxation.

These experiments along with the evidence already mentioned, strongly indicate that chromatin relaxation is most probably a downstream event after DNA damage, at least in the context of p19 induction.

### Chloroquine-mediated p19 Induction Increases the Ability of Neuro-2a Cells to Repair UV-damaged DNA

**Figure 6:**
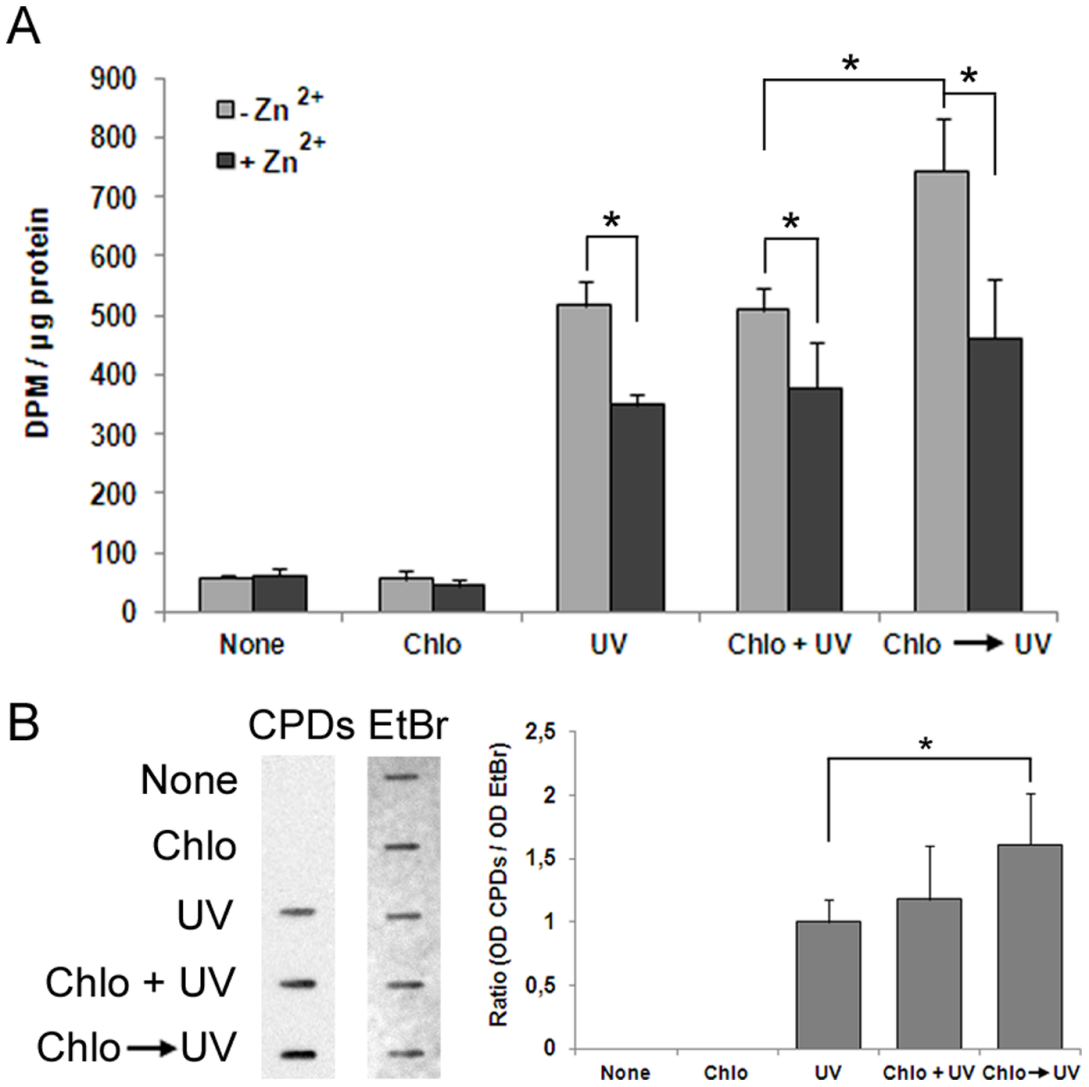
Chloroquine-mediated induction of p19 increases the ability of Neuro-2a cells to repair UV-damaged DNA. **A.** Stably transfected p19 AS Neuro-2a cells were cultured in a serum free-medium during 24 h, and then incubated with 50 µM ZnSO_4_ during 16 h. After this time, cells were treated with 100 µM chloroquine and, simultaneously (chlo+UV) or after 4 h (chlo → UV) irradiated with 40 J/m^2^ UV, and incubated with 10 µCi/ml [^3^H]thymidine for 10 h. Cell lysates were tested for unscheduled DNA synthesis assay. Bars represent the mean ± S.D. of three different experiments performed in triplicate. Student’s *t*-test was used to compare Zn^2+^-treated with non treated samples (* p<0.05) and to compare chloroquine → UV-treated with chloroquine+UV-treated from stable transfectant samples (* p<0.05). **B.** Neuro-2a cells were assayed for the presence of CPD lesions. Stably transfected p19AS Neuro-2a cells were incubated in a free-Zn^2+^ medium and treated with 100 µM chloroquine and, simultaneously (Chlo+UV) or after 4 h (chlo → UV) irradiated with 40 J/m^2^ UV. Immediately after UV irradiation, cells were harvested, DNA isolated and examined for the presence of CPD lesions by immuno-slot blot using and specific antibody. Ethidium bromide staining was used to ensure equal protein content. Figure shows a representative photograph of three independent experiments. Densitometric analysis of CPD lesions is represented in the right panel. Bars represent the mean ± S.D. of three experiments performed in triplicate. Student’s *t*-test was used to compare samples treated with choroquine → UV with samples treated with UV. (* p<0.05). Chloroquine (Chlo), EtBr (ethidium bromide).

We next asked about the physiological relevance of this mechanism. First, we hypothesized that disturbed chromatin structure would make DNA more susceptible to damage. To confront this hypothesis, we determined the DNA damage caused by UV irradiation alone or in the presence of chloroquine measured as the level of CPD lesions formed. In the latter case, chloroquine was added simultaneously with UV irradiation or 4 h before cells were irradiated (Fig. 6B). The CPD lesions observed were actually due to UV light as chloroquine by itself was unable to form such structures. Cells treated with chloroquine and UV at the same time displayed CPD levels similar to those of cells treated with UV alone. However, when the same UV dose was applied to Neuro-2a after a 4 h chloroquine treatment –a lapse sufficient to alter chromatin structure (Fig. S1)– the CPD lesions detected were significantly higher. These results strongly support our above hypothesis.

To evaluate whether p19 induction in response to distortion of chromatin structure plays a physiological role in the maintenance of genomic stability, we used a Neuro-2a cell line stably transfected with a vector harboring p19 cDNA in the reverse orientation whose expression is driven by a Zn^2+^-induced promoter. When these cells were incubated with 50 µM Zn^2+^, p19 expression was significantly diminished and its UV-mediated induction almost completely blocked (Fig. S4). Unscheduled DNA synthesis was carried out to determine the ability of these cells to repair UV-damaged DNA in the presence or in the absence of chloroquine. As previously reported, p19-deficient cells display a diminished ability to repair UV-mediated DNA damage ([21] and Fig. 6A). This result actually points out a lower capacity of DNA repair since similar levels of UV-mediated DNA damage were observed in cell lines that overexpress or underexpress p19 mRNA.

The reasoning behind the experiment was whether distortion of chromatin structure facilitates UV damage on DNA, and whether cells which express proteins, like p19, whose transcription is sensitive to chromatin defects could ameliorate the adverse consequences derived from the greater impact of genotoxicity better than cells which do not express such proteins. A significant increase in DNA repair was observed in UV-irradiated Neuro-2a p19AS cells previously treated with chloroquine with respect to UV-irradiated cells simultaneously treated with chloroquine both in the absence of Zn^2+^ (compare chloroquine→UV with UV+chloroquine, gray bars) (Fig. 6A). Conversely, cells with diminished levels of p19 displayed no significant changes in DNA repair activity in the same conditions (Fig. 6A compare chloroquine→UV with choloroquine+UV, black bars). Importantly, these results reveal that p19-deficient cells were unable to cope with the greater exposure to DNA-damaging agents like UV in a relaxed chromatin environment.

## Discussion

The maintenance of genomic integrity is of main importance to the survival and health of organisms, which are continuously exposed to genotoxic stress. Cells respond to DNA damage by activating survival pathways consisting of cell cycle checkpoints and repair mechanisms. The signal that triggers the DDR is not necessarily a direct detection of the primary DNA lesion. In contrast, in many cases it could include recognition of genotoxic intermediates or detection of abnormal chromatin structure [40], [41].

The present study uncovers the activation mechanism of p19 in response to changes in chromatin structure in a DNA damage-independent manner. The results show that p19 is a downstream target of the main DDR signaling pathway: ATM/ATR and Chk1/Chk2 kinases. In addition, ATM/ATR activation would arise without introduction of any DNA damage and suggests that these kinases could be activated immediately by modulation of chromatin condensation. In agreement with our results, it has been reported that DNA-intercalating agents such as chloroquine, inhibitors of histone deacetylases, and hypotonic conditions, which all disturb chromatin state, can lead to activation of the ATM kinase without introduction of DNA damage [14].

There are several possible ways by which chromatin defects could participate in the induction of checkpoints: a) they might make DNA more sensitive to damage, thus activating the DDR, b) the chromatin defects might cause structural changes that block replication, leading to checkpoint activation and S-phase arrest, c) chromatin defects could be a direct initiator of the checkpoint response. Since the alterations in chromatin structure triggering p19 induction were independent of DNA damage and cell cycle progression, our results are consistent with the third possibility. We cannot discard that other kinds of DNA damage distinct of double strand-breaks or CPD dimers could be caused. However, the possibility that all three chromatin modifiers assayed would induce DNA damage is very unlikely.

Our results strongly support that the transcription factor E2F1 plays a role in response to disturbed chromatin structure, motivated either by DNA damage or any other origin, connecting the ATM/ATR-Chk1/Chk2 pathway with the induction of p19 expression. In this regard, Zhang et al. reported that the small subunit of eukaryotic ribonucleotide reductase (RRM2), an enzyme complex essential for *de novo* synthesis of deoxyribonucleotides, is transcriptionally upregulated upon DNA damage and that induction dependens on E2F1 transcription factor. The authors demonstrated that, after Chk1 silencing, E2F1 expression was reduced both at the mRNA and protein levels, indicating that the regulation of RRM2 expression is mediated by Chk1-dependent upregulation of E2F1 [42]. On the other hand, it has been established that E2F1 is a physiological target of Chk2 kinase in response to DNA damage. Thus, phosphorylation by Chk2 increases E2F1 levels through an extended half life [43]. Our results are in agreement with those described above. We observed that the upregulation of E2F1 and its increased transcriptional activity in response to either DNA damage or irregular chromatin structure depend on Chk1 and Chk2 kinase activities.

Defective structures of chromatin can be produced independently of DNA damage. These alterations can have adverse consequences in the accomplishment of the diverse cellular functions that are executed on the DNA: interferences in the mechanisms of replication and transcription, abnormal recruitment of regulatory proteins, greater exposure to DNA-damaging agents, among others. It is logical to assume that the presence of this defective chromatin triggers a response similar to that of DDR in the prevention of potential damage. In this respect, the induction and activation of E2F1, which enhances mechanisms of repair and apoptosis [44], [45] and, on the other hand, the subsequent induction of p19, through its DNA repairing and antiapoptotic properties [19], [20], would lead to an adequate balance between cell death and survival signals, which would contribute to the maintenance of genomic integrity. The observation that p19-expressing cells have greater capacity to repair damaged DNA in a relaxed-chromatin context points out in this direction.

**Figure 7:**
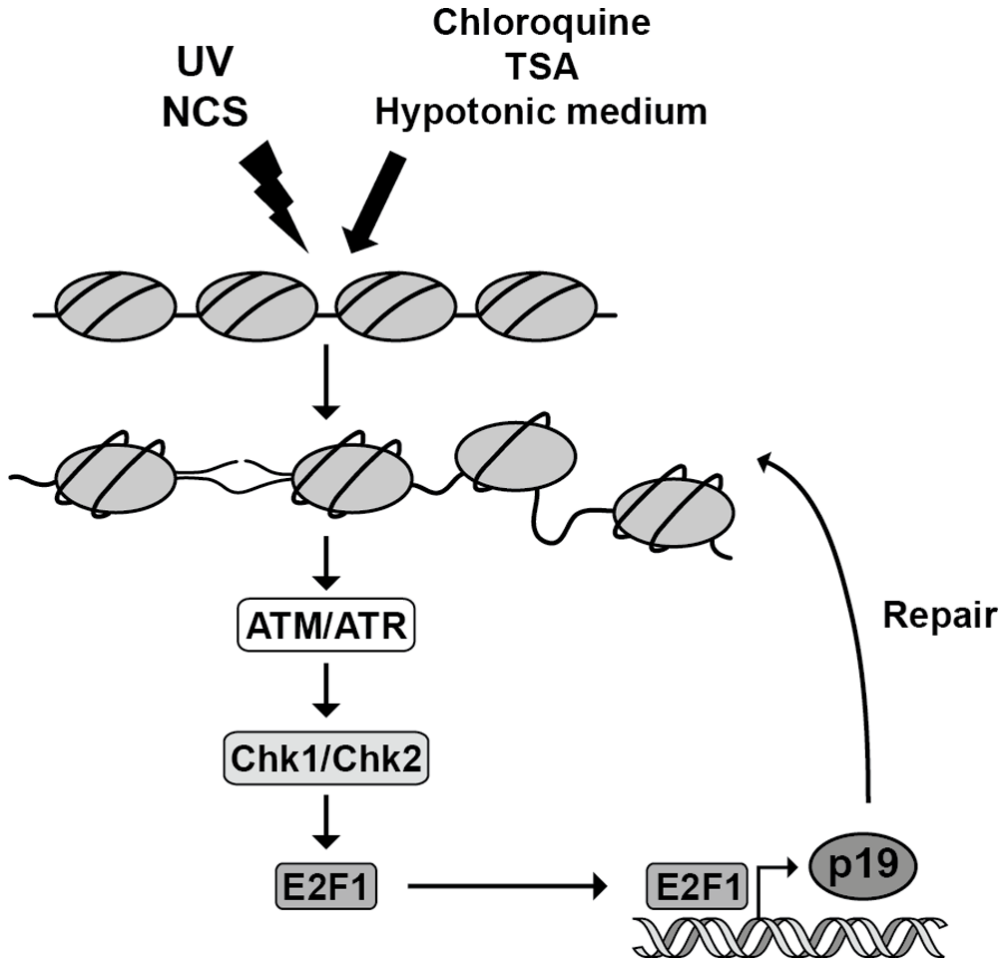
Chromatin-based defects as inducers of p19 protein. Chromatin structure defects can trigger ATM/ATR and Chk1/Chk2 activation leading to an increase in E2F1 protein levels as a result of transcriptional induction. E2F1 upregulation induces the transcription of p19 which, in turn, promotes DNA repair.

Based on these results, we propose a model that integrates chromatin-disruption events, the DDR signaling pathway and p19 (Fig. 7). According to this model, alterations in chromatin structure lead to activation of ATM/ATR kinases and the checkpoint kinases Chk1/Chk2, which in turn induce the E2F1 gene and increased levels of E2F1 transcription factor. Transcriptional activation of p19 by E2F1 would contribute to enhancing the capacity of the cells to repair DNA in case of a potential genotoxic injury.

In light of these data, we propose that an alteration in chromatin structure could initiate a checkpoint response by itself. This hypothesis implies the coexistence of two checkpoint activation pathways, one through DNA lesions and its metabolic intermediates, and the other from chromatin-based defects. The existence of these two pathways would be advantageous for the cell task directed to maintaining genomic integrity.

## Materials and Methods

### Cell Culture and Transfections

HEK293 (ATCC, CRL-1537), Neuro-2a (ATCC, CCL-131), Seckel (Coriell Cell Repositories, GM09812) and C5RO (human fibroblast line immortalized by the stable expression of telomerase) [46] cells were grown in Dulbeccós modified Eagle medium (DMEM) (Invitrogen) supplemented with 10% fetal bovine serum (FBS), 1% penicillin/streptomycin, 100 mM non-essential aminoacids, and 2 mM glutamine (Invitrogen) at 37°C in a humidified 5% CO_2_ atmosphere. SH-SY5Y cells (ATCC, CRL-2266) were grown in DMEM/F12 medium and similarly supplemented. For establishment of Neuro-2a stable clones, the pMTCB6 vector, containing p19 cDNA in the reverse orientation was used [20]. Transfections were performed using Lipofectamine™ 2000 Reagent (Invitrogen). Twenty-four hours after transfection cells were replated at low density to allow the isolation of single colonies. The clonal cell lines derived from the transfectants (p19AS and empty vector) were maintained in selective medium containing 400 µg/ml geneticin disulfate (G418, Calbiochem-Novabiochem). For metallothionein promoter induction stable transformants were treated with 50 µM ZnSO_4_ for at least 12 h. Treatment of parental Neuro-2a cells with up to 150 µM ZnS0_4_ for 12 h did not alter p19 mRNA levels.

Caffeine, KU-55933, SB-218078, and Chk2 Inhibitor were added to the medium one hour prior to the correspondent treatment. Cells were transfected with an expression vector encoding E2F1 cDNA or with a 500 nM decoy oligodeoxynucleotide harboring the E2F binding site with Lipofectamine™ 2000 Reagent (Invitrogen). Decoy sequence is as follows: 5′-ATG CGC GAA ACG CGT TTT CGC GTT TCG CGC ATA GTT TTC T-3′. Twenty four hours after transfection cells were exposed to DNA damaging or chromatin relaxing conditions. Heat shock treatments were carried out a 43°C for 1 hour in a water bathe and then cultured at 37°C in fresh DMEM supplemented with 10% fetal calf serum for the indicated times [47].

### Chromatin Relaxation

Exponentially growing cells were incubated in fresh medium containing 100 µM chloroquine or 200 nM TSA for the indicated time intervals. For hypotonic treatment, cells were incubated in hypotonic medium (phosphate buffer saline; 0.45% glucose; 1% FBS; 50 mM or 100 mM NaCl) for one hour. Then the hypotonic medium was replaced with fresh DMEM and cells were incubated for the times indicated in each case.

### DNA Damage

Exponentially growing cells were trypsinized and seeded at 50–60% confluence. Twenty four hours after plating, cells were irradiated in open-dishes with the corresponding with 40 J/m^2^ UVC dose, 254 nm (range 240–280 nm) at room temperature. Following UV-irradiation, medium was replaced and cells were incubated for the indicated time at 37°C in a 5% CO_2_ humidified incubator along times indicated in each case. Neocarzinostatin (Sigma-Aldrich) was used in some cases to induce DNA damage. This drug was added to exponentially growing cells in a final concentration of 50 ng/ml (unless otherwise indicated) for the indicated period of time.

### RNA Extraction and Northern Blot Analysis

Total cellular RNA was isolated from cultured cells as described previously [48]. Ten µg of total RNA were denatured, electrophoresed in 1% glyoxal/agarose gels, and transferred to nylon membranes (Hybond-N^+^, GE Healthcare). The membranes were sequentially hybridized with ^32^P-labeled probes as described before [19]. The membranes were scanned onto a Bio-Imaging Analyzer Fujifilm BAS-1800II. Quantification of the bands obtained was performed using ImageJ program (NIH).

### Western Blot

HEK 293 and SH-SY5Y cells lysates for immunoblotting were prepared by scraping cells into radioimmune precipitation assay buffer (1x PBS; 1% Nonidet P-40; 0.5% sodium deoxycholate; 0.1% SDS; 10 µg/ml phenylmethylsulfonylfluoride; 60 µg/ml aprotinin and 1 mM sodium orthovanadate). The lysates were centrifuged at 10,000 g for 10 min to remove cell debris. Cell lysates (20 µg) were fractionated by SDS-PAGE and thereafter blotted to a nitrocellulose membrane. Staining with Ponceau S was used to ensure equal protein content. The membrane was immunoblotted with monoclonal mouse anti-human p19 antibody (USB). The antibody was detected using horseradish peroxidase-linked goat anti-mouse IgG (Santa Cruz), visualized by the ECL detection system (Amersham-Pharmacia) and a Bio-Imaging Analyzer Fujifilm LAS-1000. Quantification of the bands obtained was performed using ImageJ program (NIH).

Total histones were purified by an acid extraction method according to manufactureŕs procedure (Upstate). Briefly, adherent cells were washed and harvested in 1 ml PBS, centrifuged at 200×g for 10 minutes and incubated on ice for 30 minutes in 5 volumes of lysis buffer (10 mM HEPES ph 7.9; 1.5 mM MgCl_2_; 10 mM KCl) with hydrochloric acid at a final concentration of 0.2 N. The acid soluble fraction containing the histones was recovered by centrifugation at 11,000 g for 10 minutes at 4°C. γH2AX was detected using a monoclonal antibody from Upstate following manufactureŕs recommendations, with a dilution 1∶1000 in TTBS buffer.

### Reporter Gene Assay

The reporter plasmids used were: p19CAT, containing 2250 bp of the human 5′-flanking region of p19 gene upstream of the chloramphenicol acetyltransferase (CAT) reporter gene in vector pBLCAT6 and p19mutCAT harboring mutations in the two E2F binding sites of p19 promoter. E2F sites in the human p19 promoter were mutated as follows: TTTCCCGC to TTTCCTAC (−630/−629 from TIS) and GCGCGACC to ATGCGACC (-685/−684). Plasmid pE2WTx4CAT, encoding the chloramphenicol acetyltransferase (CAT) reporter gene driven by an E2 core promoter and four copies of the E2F enhancer [49], was kindly provided by M. Imperiale (University of Michigan Medical School).

Cells were transfected following the standard calcium phosphate precipitation method essentially as previously described [50]. Briefly, cells seeded in 6-well dishes were transfected with 4 µg p19CAT or equal amount of the mutated version, 5 µg of pCEFL-β-galactosidase and expression vectors when indicated. Total DNA amount was adjusted to 15 µg/well with non-specific DNA carrier. After 16 h, the medium was replaced by serum-free medium, and cells were further incubated for 24 h. Cells were then harvested and CAT and β-galactosidase activities were determined as previously described [50]. CAT activity was normalized to β-galactosidase activity.

### Unscheduled DNA Synthesis

Neuro-2a p19AS cells seeded in 6-well dishes were washed with PBS and growth medium was replaced by serum-free medium which was renewed after 24 h. Inhibition of DNA semiconservative synthesis was confirmed under these conditions. Cells were treated or not with 50 µM ZnSO_4_. After 16 h, cells were incubated with 100 µM choroquine and, simultaneously or after 4 h, irradiated with 40 J/m^2^ UV and further cultured in serum free-medium with 10 µCi/ml [^3^H]thymidine. Ten hours later, cells were washed three times with cold PBS, harvested and collected at 3000 *g* for 5 min. Cells were lysed with 5% TCA for 30 min and centrifuged at 10,000 *g* for 10 min. Pellet was washed twice with cold PBS and resuspended in 1 M NaOH. The incorporated radioactivity was quantified by scintillation counting. Unscheduled DNA synthesis was expressed as dpm/µg protein.

### Cyclobutane Pyrimidine Dimers (CPD) Detection by Immuno-slot Blot Assay

The amount of thymine dimers in the DNA was measured by an immune-slot-blot assay using a CPD-specific monoclonal antibody [51]. Approximately 10^6^ Neuro-2a cells were plated into 60-mm dishes, incubated with 100 µM choroquine and, simultaneously or after 4 h, irradiated with 40 J/m^2^ UV. The cells were collected immediately after irradiation and the genomic DNA was isolated as previously described [52]. Cellular DNA was denatured in TE buffer (10 mM Tris-HCl and 1 mM EDTA, pH 7.5) by boiling for 5 min and 100 ng of each sample was spotted in triplicate onto a Hybond N^+^ membrane (GE Healthcare) using a slot blot apparatus. DNA was fixed to the membrane for 20 min on 3 MM paper soaked in 0.4 N NaOH. The membranes were blocked overnight in phosphate-buffered saline, 0.2% Tween 20 (PBS-T) containing 5% (w/v) skim milk. After washing in PBS-T, the membranes were incubated for 2 h at room temperature with a monoclonal antibody specific for thymine dimers (Kamiya Biomedical) at a dilution of 1/2000 in blocking buffer. The antibody was detected using horseradish peroxidase-linked goat anti-mouse IgG (Santa Cruz), visualized by the ECL detection system (Amersham-Pharmacia) and a Bio-Imaging Analyzer Fujifilm LAS-1000. Quantification was performed using ImageJ program (NIH). The membranes were stained with ethidium bromide (10 µg/ml) and 1% methylene blue in order to ensure equal amounts of loaded DNA and this quantification was used to relativized the CPD lesions assessed.

## Supporting Information

Figure S1 **Cloroquine, TSA and hypotonic medium increased MNase accessibility of chromatin.** HEK-293 cells were incubated with 100 µM chloroquine (**A**) or 200 nM TSA (**B**) or hypotonic medium (50 mM NaCl) (**C**) as indicated. After 4 h whole nuclei were isolated and incubated with 2 U/ml MNase for the indicated times. Total genomic DNA was purified and the pattern of DNA digestion was analyzed by electrophoresis as described in materials and methods section. Each figure shows a representative gel of three independent experiments with similar results. Choroquine (Chlo), microccocal nuclease (MNase), hypotonic (Hypo) and isotonic (Iso) medium, markers (M).
(TIF)

Figure S2 **p19 is the only member of INK4 family that is induced by chromatin relaxation.** HEK-293 cells were exposed to 100 µM chloroquine, 200 nM TSA or hypotonic medium (50 mM NaCl) for the indicated times. Total RNA (10 µg) extracted from cells at the indicated times were subjected to northern blot analysis with the ^32^P-labeled probes specified at the right margin. Figure shows a representative autoradiograph of three independent experiments with similar results. Chloroquine (chlo), hypotonic medium (hypo), β-tubulin (β-tub), neocarzinostatin (NCS).
(TIF)

Figure S3 **Induction of p19 by chromatin modifying agents is independent of double strand DNA damage and cell cycle arrest. A.** HEK-293 cells were incubated with 100 µM chloroquine or 200 nM TSA or hypotonic medium (50 mM NaCl) as indicated. After 4 h histone proteins were purified by an acid extraction protocol. The level of H2AX phosphorylation (γH2AX) was assessed by western blot. Cells incubate with 1 µM camptothecin was used as a positive control. Histone H3 was used as a loading control. Figure shows a representative western of three independent experiments with similar results **B.** HEK-293 cells were incubated with 100 µM chloroquine or 200 nM trichostatin A or hypotonic medium as indicated. After 4 h cells were harvested and subjected to flow cytometric cell cycle analysis. Mimosine (200 µM) was used as a G1/S boundary arrest positive control. Bars represent de mean ± S.D. of four independent experiments performed in duplicate. Student’s t test was used to compare % of cells in G1 and S phases from 24 h mimosine treated cells with 4 h mimosine treated cells (* p<0.05). Camptothecin (CT), chloroquine (chlo), hypotonic medium (hypo).
(TIF)

Figure S4 **Diminished expression of p19 mRNA in Zn^2+^-treated Neuro-2a p19AS cells.** Total RNA was extracted from 50 µM ZnS0_4_ treated and/or 40 J/m^2^ UV irradiated stably transfected p19AS cells and subjected to northern blot analysis using a ^32^P-labeled probe specific for p19 mRNA and reprobed for β-tubulin (β-tub) mRNA. Figure shows a representative autoradiograph of three independent experiments with similar results. Densitometric analysis of p19 is represented in the right panel. Bars represent the mean ± S.D. of three experiments. Student’s *t*-test was used to compare treated and non-treated samples (* p<0.05, at least).
(TIF)

Materials and Methods S1 (DOC)
