# Supplementary figures and images for: Chromatin Relaxation-Mediated Induction of p19INK4d Increases the Ability of Cells to Repair Damaged DNA
Source: PLoS One. 2013 Apr 12;8(4):e61143. doi: 10.1371/journal.pone.0061143 (PMC3625165; doi:10.1371/journal.pone.0061143)

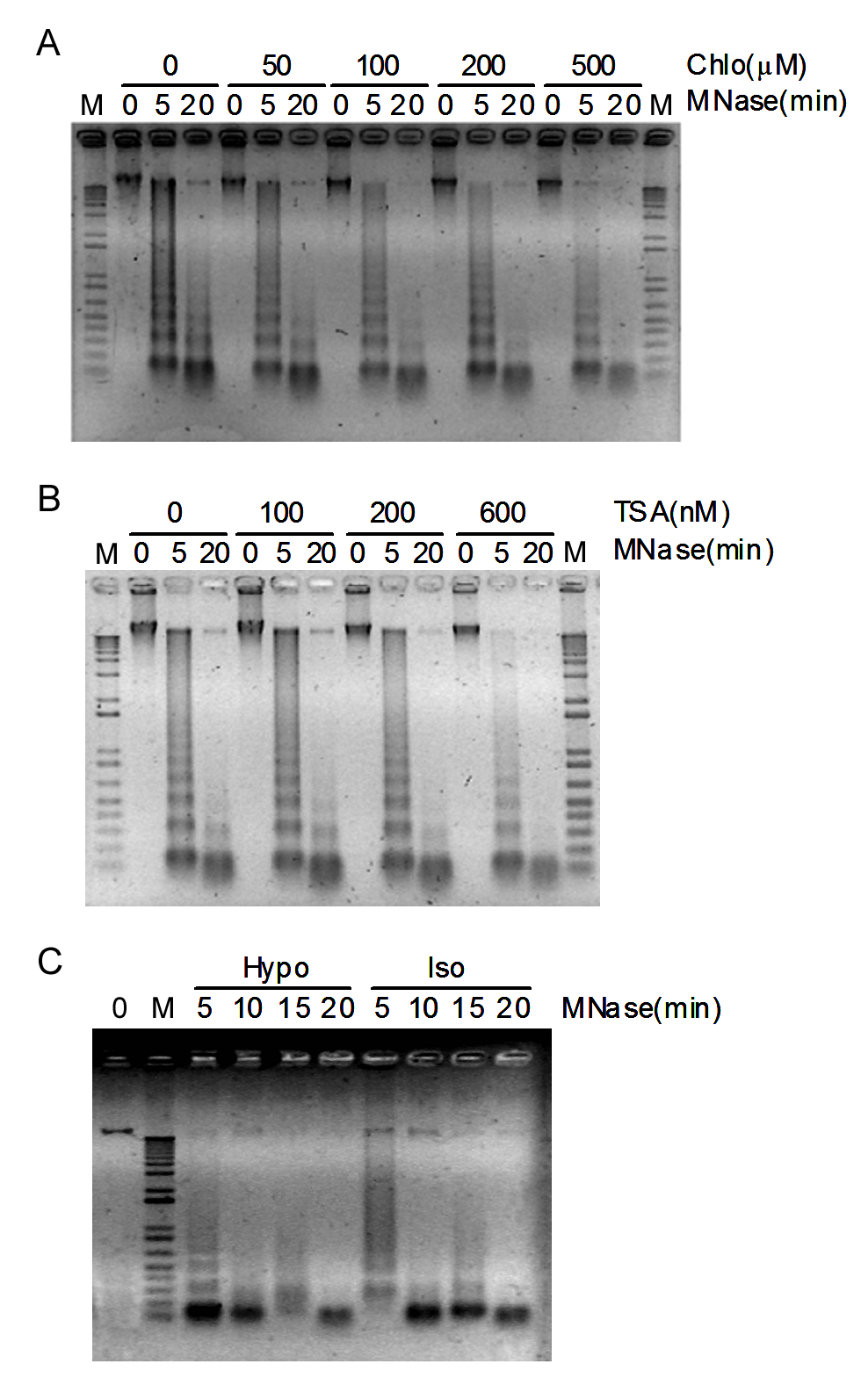

Supplement: Figure S1 — Cloroquine, TSA and hypotonic medium increased MNase accessibility of chromatin. HEK-293 cells were incubated with 100 µM chloroquine (A) or 200 nM TSA (B) or hypotonic medium (50 mM NaCl) (C) as indicated. After 4 h whole nuclei were isolated and incubated with 2 U/ml MNase for the indicated times. Total genomic DNA was purified and the pattern of DNA digestion was analyzed by electrophoresis as described in materials and methods section. Each figure shows a representative gel of three independent experiments with similar results. Choroquine (Chlo), microccocal nuclease (MNase), hypotonic (Hypo) and isotonic (Iso) medium, markers (M). (TIF) [file pone.0061143.s001.tif]

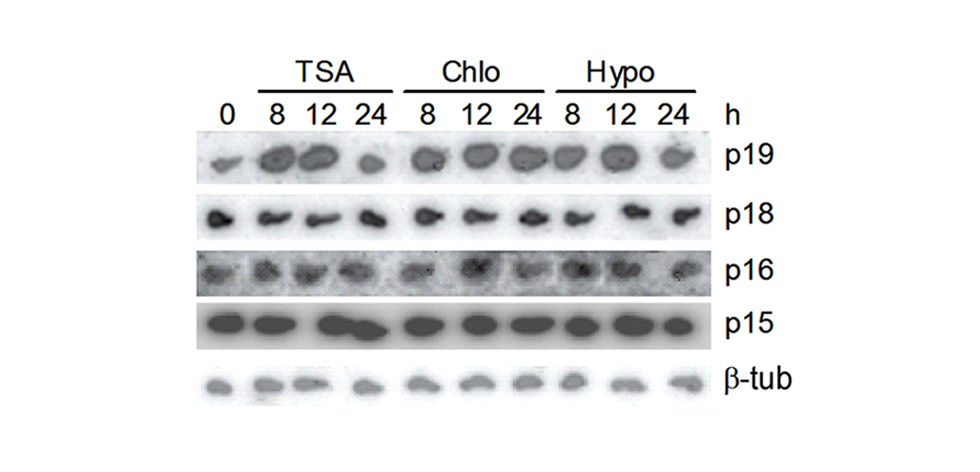

Supplement: Figure S2 — p19 is the only member of INK4 family that is induced by chromatin relaxation. HEK-293 cells were exposed to 100 µM chloroquine, 200 nM TSA or hypotonic medium (50 mM NaCl) for the indicated times. Total RNA (10 µg) extracted from cells at the indicated times were subjected to northern blot analysis with the 32P-labeled probes specified at the right margin. Figure shows a representative autoradiograph of three independent experiments with similar results. Chloroquine (chlo), hypotonic medium (hypo), β-tubulin (β-tub), neocarzinostatin (NCS). (TIF) [file pone.0061143.s002.tif]

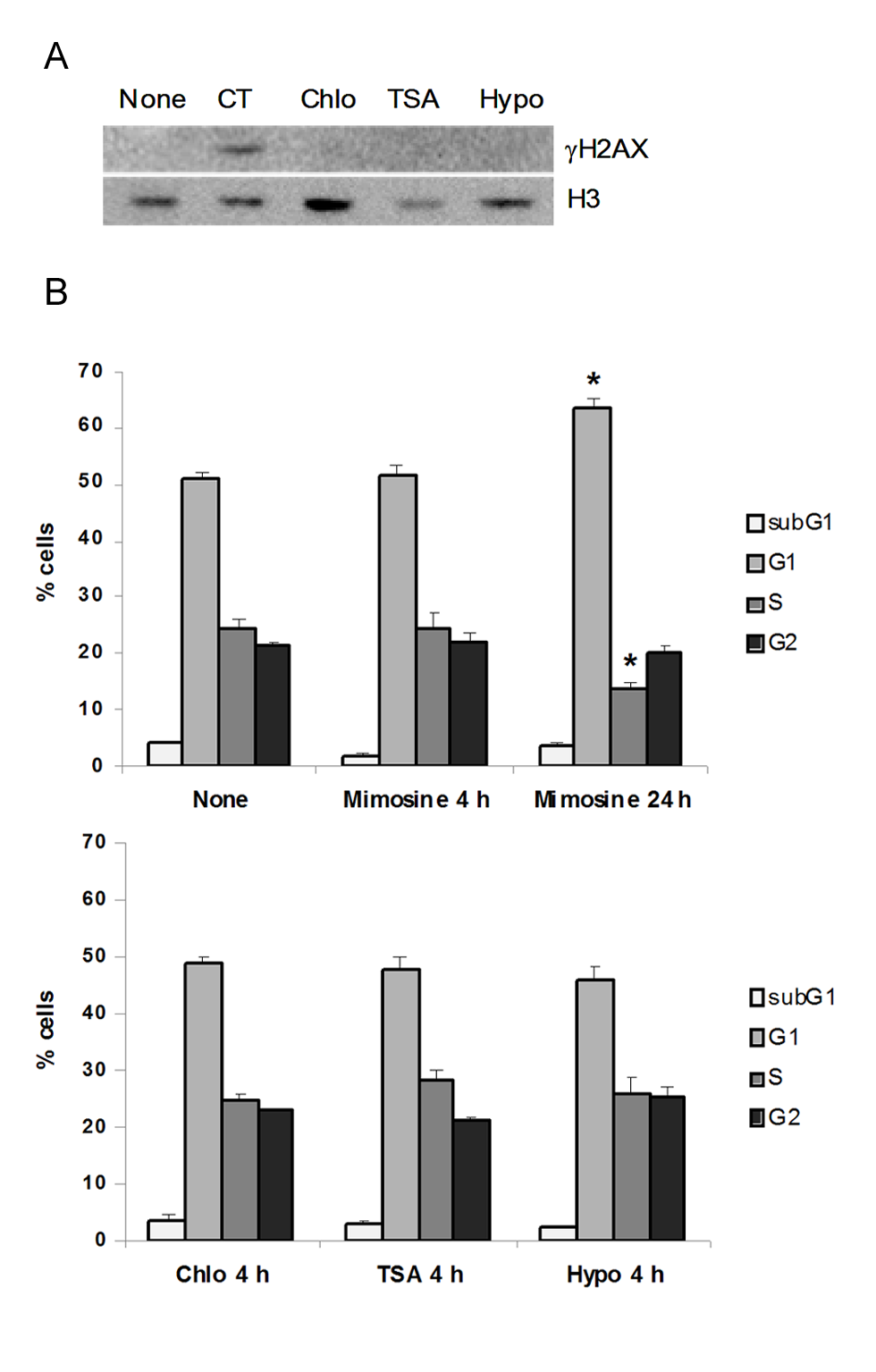

Supplement: Figure S3 — Induction of p19 by chromatin modifying agents is independent of double strand DNA damage and cell cycle arrest. A. HEK-293 cells were incubated with 100 µM chloroquine or 200 nM TSA or hypotonic medium (50 mM NaCl) as indicated. After 4 h histone proteins were purified by an acid extraction protocol. The level of H2AX phosphorylation (γH2AX) was assessed by western blot. Cells incubate with 1 µM camptothecin was used as a positive control. Histone H3 was used as a loading control. Figure shows a representative western of three independent experiments with similar results B. HEK-293 cells were incubated with 100 µM chloroquine or 200 nM trichostatin A or hypotonic medium as indicated. After 4 h cells were harvested and subjected to flow cytometric cell cycle analysis. Mimosine (200 µM) was used as a G1/S boundary arrest positive control. Bars represent de mean ± S.D. of four independent experiments performed in duplicate. Student’s t test was used to compare % of cells in G1 and S phases from 24 h mimosine treated cells with 4 h mimosine treated cells (* p<0.05). Camptothecin (CT), chloroquine (chlo), hypotonic medium (hypo). (TIF) [file pone.0061143.s003.tif]

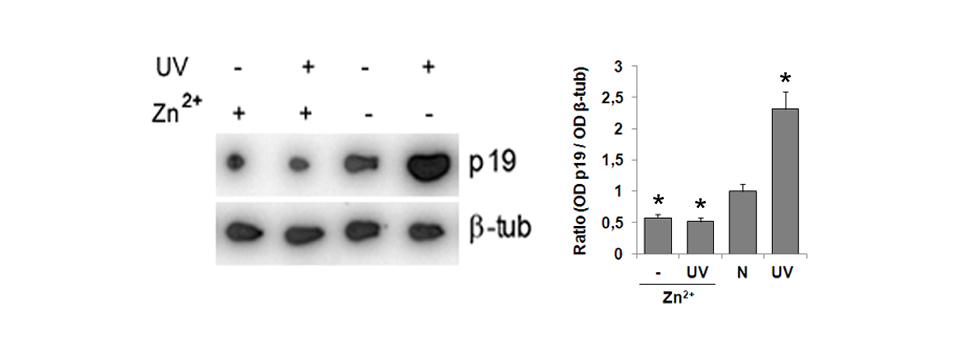

Supplement: Figure S4 — Diminished expression of p19 mRNA in Zn2+-treated Neuro-2a p19AS cells. Total RNA was extracted from 50 µM ZnS04 treated and/or 40 J/m2 UV irradiated stably transfected p19AS cells and subjected to northern blot analysis using a 32P-labeled probe specific for p19 mRNA and reprobed for β-tubulin (β-tub) mRNA. Figure shows a representative autoradiograph of three independent experiments with similar results. Densitometric analysis of p19 is represented in the right panel. Bars represent the mean ± S.D. of three experiments. Student’s t-test was used to compare treated and non-treated samples (* p<0.05, at least). (TIF) [file pone.0061143.s004.tif]
