# Supplementary material for: Chromatin Relaxation-Mediated Induction of p19INK4d Increases the Ability of Cells to Repair Damaged DNA
Source: PLoS One. 2013 Apr 12;8(4):e61143. doi: 10.1371/journal.pone.0061143 (PMC3625165; doi:10.1371/journal.pone.0061143)
Supplement: Materials and Methods S1 — (DOC) [file pone.0061143.s005.doc]

**SUPPORTING INFORMATION**

**Materials and Methods S1**

***Micrococcal nuclease digestion***

Nuclease digestion was performed as previously described [52]. Briefly, adherent cells were washed and harvested in 1 ml PBS, centrifuged at 200 x g for 10 minutes and resuspended in 3 volumes of RSB buffer (10 mM Tris-HCl pH 7,5; 10 mM NaCl; 1 mM MgCl_2_; 250 mM Sucrose; 0,1 mM PMSF) with 0,5 % NP-40 and incubated on ice for 5 minutes. Nuclear pellet was obtained by centrifugation and resuspended in RSB buffer. Nuclear integrity was assessed by Trypan Blue staining and nuclei solution was diluted in RSB buffer containing 1 mM CaCl_2_ at a final concentration of 50,000 nuclei/μl. Reaction volumes of 200 μl were preincubated at 37 °C for 10 minutes and MNasa mix (2 U/ml) was added for the indicated time intervals, after which 1 volume of stop solution (20 mM EDTA pH 8; 1% SDS) was added to the reaction to stop the digestion. DNA was purified by standard procedures and electrophoresis was carried out in 1% agarose gels.

***Flow cytometry analysis***

Cells were washed and harvested in 1 ml PBS containing 10% fetal calf serum. Cellular pellet was resuspended in 100 µl of PBS, fixed by drop wise addition of 1 ml of methanol and rested overnight at 4 °C. The fixed cells were then centrifuged and resuspended in 200 µl of RNase A (250 µg/ml) and incubated for 30 min at 37 °C. Cells were centrifuged and resuspended in 200 µl of 50 µg/ml propidium iodide. Cells were then analyzed for DNA content by flow cytometry on a FACScan (BD, San Jose, CA). Data were analyzed using the computer program WinMDI (version 2.8).
